# Two cases of idiopathic steroid-resistant nephrotic syndrome complicated with thrombotic microangiopathy

**DOI:** 10.1186/s12882-020-01985-5

**Published:** 2020-08-03

**Authors:** Kentaro Nishi, Mai Sato, Masao Ogura, Mika Okutsu, Kenji Ishikura, Koichi Kamei

**Affiliations:** 1 Division of Nephrology and Rheumatology, National Center for Child Health and Development, 2-10-1 Okura, Setagaya-ku, Tokyo, 157-8535 Japan; 2 Department of Pediatrics, Tokyo Medical and Dental University, 1-5-45 Yushima, Bunkyo-ku, Tokyo, 113-8510 Japan; 3 Department of Pediatrics, Kitasato University School of Medicine, 1-15-1, Kitazato, Minami-ku, Sagamihara-shi, Kanagawa, 252-0375 Japan

**Keywords:** Steroid-resistant nephrotic syndrome (SRNS), Thrombotic microangiopathy (TMA), Hypertension, Acute kidney injury (AKI)

## Abstract

**Background:**

Thrombotic microangiopathy (TMA) is a histopathological entity associated with microangiopathic hemolytic anemia, thrombocytopenia, and end-organ ischemic damage. Although TMA is caused by various diseases, there have been few reports regarding children with idiopathic nephrotic syndrome (NS) and TMA. Here we report two 1-year-old infants with steroid-resistant NS (SRNS) who presented with severe hypertension, acute kidney injury (AKI), and TMA.

**Case presentation:**

The diagnosis of NS was complicated with anemia, AKI, and hypertension. Maximum blood pressure was 150/70 mmHg in Case 1 and 136/86 mmHg in Case 2. There was no thrombocytopenia during their clinical course in both cases. Renal biopsy showed the features of TMA, including endothelial cell swelling, capillarectasia or marked mesangiolysis, along with mesangial proliferation in Case 1 and TMA with minor glomerular abnormalities in Case 2. Hemolytic uremic syndrome, thrombotic thrombocytopenic purpura, and secondary TMA other than that caused by hypertension were excluded. Oral prednisolone therapy, frequent infusion of albumin and diuretics, and multiple anti-hypertensive drugs were initiated. Blood pressure was controlled after 6 and 7 days from initiation of multiple anti-hypertensive drugs and lisinopril was added due to persistent mild proteinuria and mild hypertension after improvement of renal function in both cases. Proteinuria resolved completely 4 months after admission with daily oral prednisolone for 4 weeks followed by alternative daily oral prednisolone for 4 weeks in Case 1. Proteinuria resolved completely 10 months after admission with initial prednisolone treatment for 4 weeks followed by cyclosporine A and intravenous methylprednisolone pulse therapy in Case 2. The follow-up biopsy showed no TMA findings in both patients. Because the patient in Case 1 subsequently developed frequent relapsing NS, cyclosporine A was commenced after the second biopsy and he did not have any flares for 2 years. Renal function was normal in Case 1 and mildly decreased in Case 2 at last follow-up (creatinine-eGFR of 136.2 mL/min/cm^2^ in Case 1 and 79.5 mL/min/cm^2^ in Case 2).

**Conclusion:**

Severe hypertension and AKI can be signs of TMA in patients with SRNS. Strict anti-hypertensive therapy might improve renal outcomes.

## Background

Thrombotic microangiopathy (TMA) is defined pathologically by endothelial injury and thrombi formation in microvasculature. TMA is caused by various diseases and conditions that include hemolytic uremic syndrome (HUS), thrombotic thrombocytopenic purpura (TTP), atypical HUS (aHUS), malignant hypertension, infection, malignancy, and medications [1]. However, there have been very few case reports of idiopathic nephrotic syndrome (NS) complicated with TMA. In addition, its pathogenesis and management are not completely clarified [2].

Approximately 10–20% of the patients with NS do not respond to steroid therapy (steroid-resistant NS, SRNS) [3]. SRNS is defined by the absence of remission after 1 month of daily prednisone therapy at a dose of 60 mg/m^2^ per day [3]. The common histological diagnosis of SRNS includes focal segmental glomerulosclerosis (FSGS), minor glomerular abnormalities (MGA), and mesangial proliferation [3]. Benz et al. reported a 12-year-old female patient with FSGS, complicated with TMA, and followed by progression to end-stage renal disease (ESRD) [2]. Most patients with TMA who presented with renal involvement have poor life and renal outcomes [4, 5].

Here we report the effectiveness of strict anti-hypertensive therapy for two 1-year-old infants with SRNS, complicated with TMA, who presented with severe hypertension and acute kidney injury (AKI).

## Case presentation

### Case 1

A boy 1 year and 5 months old developed periorbital edema and gross hematuria. He was admitted to a local hospital with a diagnosis of idiopathic NS. On the following day, AKI and hypertension were noted and he was referred to our institution. His past medical history and family history were unremarkable. On admission, severe bilateral edema of eyelids and legs was noted. Physical examination revealed severe bilateral edema and urinalysis demonstrated prominent proteinuria (urinary protein/creatinine ratio [UP/Cr] 31.6 g/gCr), hematuria (sediment RBC > 100/HPF) and hypercholesteremia (total cholesterol 379 mg/dL) at the time of admission. His body weight was 16.9 kg, which had increased by 5.1 kg from his usual weight. His blood pressure was 112/70 mmHg. Urinary output was 0.6 mL/kg/h. Laboratory examination revealed hypoalbuminemia (serum albumin 1.0 g/dL), renal insufficiency (creatinine 0.61 mg/dL, creatinine-eGFR 43.7 mL/min/cm^2^, urea 28.2 mg/dL), hyperkalemia (potassium 6.7 mEq/L), anemia (Hb 9.6 g/dL, MCV 80.6 fL, MCH 25.8 pg, MCHC 31.9 g/dL), hyperlipidemia (triglycerides 709 mg/dL) and increased total cholesterol (total cholesterol 428 mg/dL). Thrombocyte count (30.8 × 10^4^/μL), lactate dehydrogenase (291 U/L), total bilirubin (0.31 mg/dL), and aspartate aminotransferase values (28 U/L) were normal. Iron level was 27 μg/dL, TIBC 113 μg/dL, and ferritin 63.4 ng/mL. Reticulocyte count and the Coombs test were not performed. Complement, ASO, ASK, PR3-ANCA, MP3-ANCA, anti-GBM antibodies, antinuclear antibody, and anti–double-stranded DNA immunoglobulin demonstrated no abnormal findings. Hypertension and renal insufficiency progressed gradually (blood pressure of 150/70 mmHg and blood creatinine of 0.85 mg/dL on hospital day 6). Blood smear examination revealed schistocytes from hospital day 6 to hospital day 30. Thrombocyte count, lactate dehydrogenase, bilirubin, and aspartate aminotransferase values were normal during the clinical course.

**Fig. 1:**
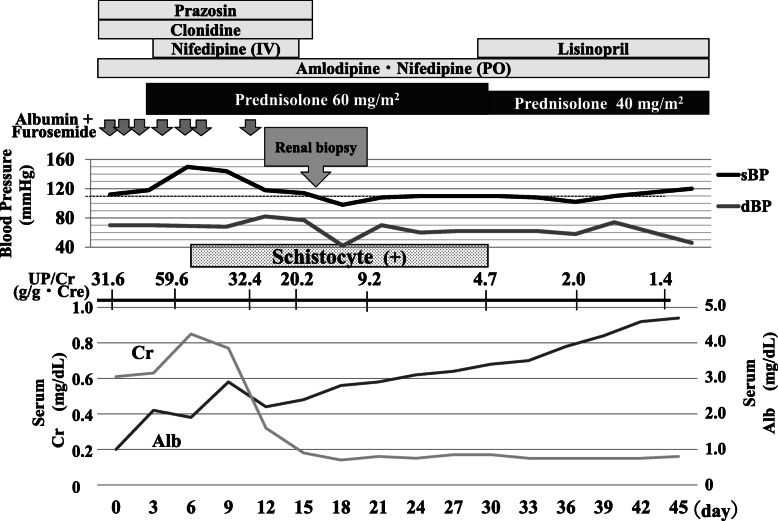
Clinical course in Case 1. Clinical course after admission in Case 1. sBP, systolic blood pressure; dBP, diastolic blood pressure; Cr, creatinine; Alb, albumin; UP/Cr, urinary protein/creatinine ratio

**Fig. 2:**
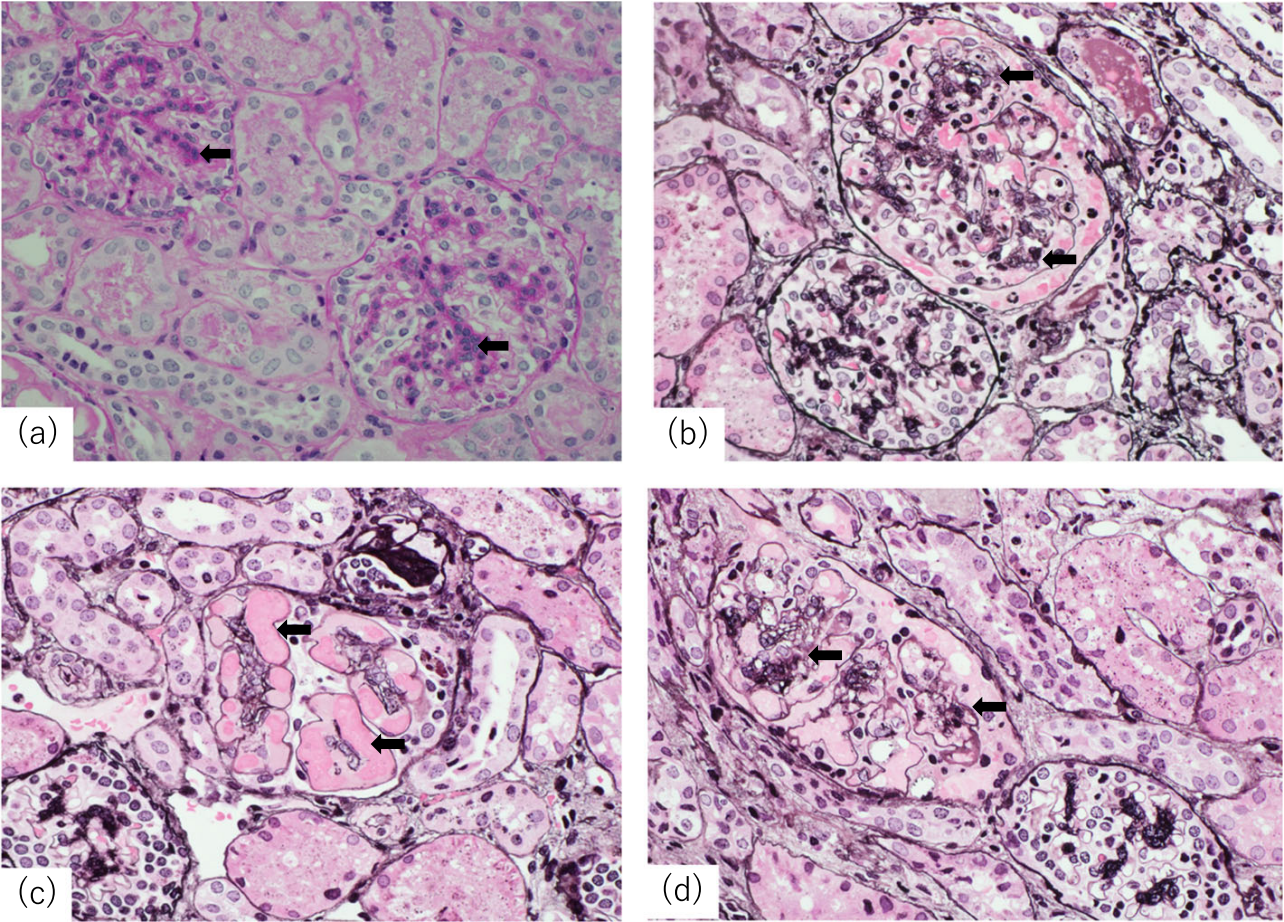
Pathology of renal biopsy in Case 1. **a** Mild-to-moderate diffuse mesangial proliferation, PAS, × 400 (**b**) TMA with mild endothelial cell swelling and marked mesangiolysis, PAM, × 400 (**c**) Capillarectasia with mesangiolysis, PAM, × 400 (**d**) Endothelial cell swelling, PAM, × 400. PAM, periodic acid methenamine silver stain; PAS, periodic acid Schiff stain; TMA, thrombotic microangiopathy

The clinical course after admission is shown in Fig. 1. The patient was treated for idiopathic NS with daily oral prednisolone for 4 weeks at a dose of 60 mg/m^2^ body surface area, then tapered. However, he could not achieve remission and his disease was diagnosed as SRNS. Frequent administration of albumin and diuretics was required to prevent nephrotic crisis. Severe hypertension was treated with multiple anti-hypertensive drugs (intravenous nicardipine and oral amlodipine, nifedipine, clonidine, and prazosin). Systolic blood pressure was controlled after 6 days from initiation of multiple anti-hypertensive drugs. No acute end-organ damage, such as hypertensive encephalopathy, or ocular complications secondary to hypertension developed. An initial renal biopsy was performed on hospital day 15. The biopsy contained 50 glomeruli; diffuse mild-to-moderate mesangial proliferation, endothelial cell swelling, and marked mesangiolysis indicated TMA (Fig. 2). There was no membranoproliferative pattern including a double contour of the glomerular basement membrane. There were no obvious abnormal findings of arteries and arterioles. Immunofluorescence findings showed the following: IgG (−), IgA (−), IgM (−), C1q (−), C3 (−), C4 (−), and fibrinogen (+). Electron microscopic findings showed foot process effacement and absence of dense deposit. Stool Shiga toxin and culture were negative. Serum ADAMTS13 activity was 95.8%. Coombs tests were negative. Serum haptoglobin was undetectable (< 10 mg/dL). The patient did not have any evidence of infection, malignancy, or autoimmune disease. There were no mutations in the genes with known pathogenic mutations associated with aHUS (CFH, CFI, MCP, C3, CFB, DGKE, and THBD) and the pathogenic mutations associated with FSGS (CD2AP, NPHS1, NPHS2, PLCE1, SMARCAL1, LAMB2, SCARB2, COQ2, COQ6, ITGA3, ITGB4, GLEPP1, MYO1E, ARHGDIA, ADCK4, TTC21B, NUP93, NUP107, NUP205, NUP85, NUP133, NUP160, CRB2, CUBN, EMP2, FAT1, KANK1, KANK2, KANK4, PDSS2, PTPRO, XPO5, ACTN4, ANLN, ARHGAP24, INF2, LMX1B, MYH9, PAX2, TRPC6, WT1, WDR73, MAGI2, AVIL, TNS2, DLC1, CDK20, ITSN1, SGPL1, LMNA, LAMA5, GON7, LAGE3, OSGEP, TPRKB, TP53RK, NUP133, WDR4, GAPVD1, and ANKFY).

Although renal function and hypoalbuminemia improved gradually after 20 days from the start of prednisolone, massive proteinuria persisted. Therefore, we added lisinopril at hospital day 28. Four months after start of treatment, proteinuria resolved completely without cyclosporine A. However, he subsequently developed frequent relapsing NS. A second biopsy which was performed 7 months after the onset of SRNS contained 75 glomeruli; four glomeruli showed mild mesangial hypercellularity. There were no findings of TMA. We started cyclosporine A after the second biopsy and he did not have any flares for 2 years. During the entire course, the minimums of his hemoglobin and platelets were 7.0 g/dL (on hospital day 9) and 18.3 × 10^4^ /μL (on hospital day 3). During the next 2 years, he maintained normal renal function (creatinine level 0.27 mg/dL, creatinine-eGFR 136.2 mL/min/cm^2^) and normal blood pressure without taking anti-hypertensive drugs.

### Case 2

A girl 1 year and 2 months old was referred and admitted to our institution with prominent proteinuria (UP/Cr 27.7 g/gCr) due to idiopathic NS. Her past medical history and family history were unremarkable. On admission, severe bilateral edema of eyelids and legs was noted. Her blood pressure was 110/60 mmHg. Laboratory examination revealed hypoalbuminemia (serum albumin 1.5 g/dL), renal insufficiency (creatinine level 0.74 mg/dL, creatinine-eGFR 49.7 mL/min/cm^2^, urea 63.1 mg/dL), anemia (Hb 8.7 g/dL, MCV 87.1 fL, MCH 28.9 pg, MCHC 33.2 g/dL), hyperlipidemia (triglycerides 1039 mg/dL), and increased total cholesterol (total cholesterol 582 mg/dL). Thrombocyte count was 64.7 × 10^4^/μL, lactate dehydrogenase was 461 U/L, total bilirubin was 0.69 mg/dL, and aspartate aminotransferase was 34 U/L.

On hospital day 2, prednisolone (60 mg/m^2^ body surface area) was initiated. Frequent administration of albumin and diuretics (furosemide, hydrochlorothiazide, spironolactone, carperitide) was required. As she could not achieve remission at 4 weeks of prednisolone therapy, her disease was diagnosed as SRNS. Cyclosporine A and intravenous methylprednisolone pulse therapy were commenced 4 weeks after initiating prednisolone. As her blood pressure was high from hospital day 20, we administered anti-hypertensive drugs (intravenous nicardipine and oral amlodipine, nifedipine, and prazosin). The maximum blood pressure during the clinical course was 136/86 mmHg. Systolic blood pressure was controlled after 7 days of initiating multiple anti-hypertensive drugs. She did not develop target organ complications caused by hypertension. Complement, PR3-ANCA, MP3-ANCA, anti-GBM antibodies, antinuclear antibody, and anti–double-stranded DNA immunoglobulin were normal and Coombs tests were negative. Renal biopsy at 5 weeks after start of therapy contained 30 glomeruli and the pathological diagnosis was MGA. Five glomeruli showed capillarectasia and mesangiolysis, indicating TMA. There was no membranoproliferative pattern including a double contour of the glomerular basement membrane. There were no obvious abnormal findings of arteries and arterioles. There were the findings of acute tubular damage with obstructive nephropathy and nephrocalcinosis. Immunofluorescence findings showed the following: IgG (−), IgA (−), IgM (++), C1q (+), C3 (+), C4 (+), and fibrinogen (+). Electron microscopic findings showed foot process effacement and absence of dense deposit. Schistocytes were not present. Serum haptoglobin and ADAMTS13 were not measured. Thrombocyte counts were normal during the clinical course. Lactate dehydrogenase level was 824 U/L, bilirubin was 0.74 mg/dL, and aspartate aminotransferase was 42 U/L. There was no pathogenic mutation associated with FSGS or TMA. The patient did not have any evidence of HUS, TTP, aHUS, infection, malignancy, or autoimmune disease.

Seven weeks after admission, serum albumin was above 2.5 g/dL and renal function improved. Five months after admission, lisinopril was added due to proteinuria and mild hypertension. After lisinopril, proteinuria and blood pressure had gradually decreased. Ten months after admission, proteinuria resolved completely with seven courses of intravenous methylprednisolone pulse. During the entire course, the minimums of her hemoglobin and platelets were 7.3 g/dL (on hospital day 2) and 22.1 × 10^4^ /μL (on hospital day 9). Her second biopsy showed MGA and disappearance of findings of TMA 2 years after starting treatment. At that time, her renal function testing revealed a mild reduction in GFR (creatinine level 0.38 mg/dL, creatinine-eGFR 79.5 mL/min/cm^2^). She is currently on cyclosporine A and amlodipine.

## Discussion and conclusions

We report two patients with SRNS who presented with severe hypertension, AKI, and TMA. Although the precise cause of TMA was unclear in our cases, we considered it to be hypertension-induced TMA. Strict multiple anti-hypertensive therapy might have improved the patients’ renal outcomes.

Although the exact etiology of TMA in the present cases cannot be completely determined, we believe that these two cases are hypertension-induced TMA. Both our cases developed severe hypertension. The maximum blood pressures of cases 1 and 2 were 150/70 and 136/86 mmHg, which are 34 and 23 mmHg higher than the value of stage-2 hypertension (95th percentile + 12 mmHg), respectively [6]. We excluded HUS, TTP, and secondary TMA by investigation of aHUS-associated mutations such as CFH, CFI, MCP, C3, CFB, DGKE, and THBD. Cyclosporine A is a medicine that can lead to TMA. TMA appeared before starting cyclosporine in Case 1. Although TMA appeared after starting cyclosporine, TMA improved without withdrawal of this drug in Case 2. Therefore, the contributory effect of cyclosporine to TMA was very unlikely in our cases. Although immunofluorescence findings in Case 2 showed positive for IgM, C1q, C3, and C4, there was no electron-dense deposit. Moreover, there was no familial history associated with aHUS.

TMA due to malignant hypertension was the most common clinical disorder (56.0%) in renal biopsy-proven TMA patients [4]. The pathophysiological mechanisms underlying TMA in malignant hypertension are not well understood. Mathew et al. reported that endothelial injury in TMA was the result of increased shear stress, toxins, and/or dysregulated complement activation [7]. Benz et al. reported a 12-year-old female patient with former steroid-dependent NS who presented with severe hypertension, renal failure, and the histological pattern of TMA [2]. Unfortunately, she progressed to ESRD. Our two cases had SRNS, AKI, and severe hypertension, which were similar to this previous case. In Case 1, the schistocytes appeared after the onset of AKI and hypertension, and kidney dysfunction had not gotten worse since schistocytes appeared. Therefore, we presumed that TMA did not induce hypertension and AKI, but rather that hypertension mainly led to TMA. In addition, a vicious cycle may be created by TMA and hypertension [8].

There is a relationship between hypertension and NS [9, 10]. In a Japanese nationwide observational study, 10.8% of new-onset NS patients experienced hypertension requiring treatment [11]. In particular, the patients with SRNS had a much higher prevalence of hypertension compared to the patients with steroid-sensitive NS (66.7 and 14.3%, respectively) [10]. The etiology of hypertension in NS is multifactorial and complex [9]. It was reported that 24% of patients with NS experienced severe AKI, and hypertension was also significantly related to AKI [11]. In our cases and the previously reported case, the patients developed severe NS with AKI and required frequent administration of albumin and diuretics. These can cause fluid shifts, sodium retention, loss of GFR, and a feed-forward loop between albuminuria and blood pressure.

Our cases did not show thrombocytopenia. It was reported that patients with TMA due to hypertension tend to have a low incidence of thrombocytopenia, anemia, and elevated LDH [5]. This report supports the diagnosis of hypertension-induced TMA in our cases. On the other hand, Timmermans et al. reported that some patients with clinically diagnosed hypertension-induced TMA had identified genetic alternative pathway abnormalities [12, 13]. This is particularly the case in patients presenting without profound hemolysis and/or thrombocytopenia [12].

There are several limitations regarding the diagnosis of hypertension-induced TMA in the present report. First, screening for alternative pathway abnormalities was inadequate because some gene mutations, such as CD46 and factor H antibodies, were not checked. Second, we were unable to confirm serum haptoglobin and ADAMTS13 in Case 2.

The outcome of TMA diagnosed by renal biopsy is usually poor. Yu et al. reported 109 renal biopsy-proven TMA patients, including 61 patients in which the cause of TMA was malignant hypertension [4]. Of them, eight patients died, 17 patients had double levels of serum creatinine, and 44 had ESRD at follow-up [4]. Renal function test results in our patients at last follow-up were creatinine-eGFR values of 136.2 mL/min/cm^2^ (Case 1) and 79.5 mL/min/cm^2^ (Case 2) and the follow-up biopsies showed no TMA findings. Good renal outcomes may have resulted because we conducted early and strict anti-hypertensive therapy, including intravenous administration, in our cases.

In conclusion, severe hypertension with AKI can be a sign of TMA in patients with SRNS. Strict anti-hypertensive therapy might improve renal outcomes.

## Data Availability

The datasets used and/or analyzed during the current study are available from the corresponding author upon reasonable request.

## Abbreviations

TMA: Thrombotic microangiopathy
HUS: Hemolytic uremic syndrome
TTP: Thrombotic thrombocytopenic purpura
aHUS: Atypical hemolytic uremic syndrome
NS: Nephrotic syndrome
SRNS: Steroid-resistant nephrotic syndrome
FSGS: Focal segmental glomerular sclerosis
ESRD: End-stage renal disease
AKI: Acute kidney injury
UP/Cr: Urinary protein/creatinine ratio
